# Gene specific-loci quantitative and single-base resolution analysis of 5-formylcytosine by compound-mediated polymerase chain reaction†
†Electronic supplementary information (ESI) available. See DOI: 10.1039/c8sc00493e


**DOI:** 10.1039/c8sc00493e

**Published:** 2018-03-19

**Authors:** Yafen Wang, Chaoxing Liu, Xiong Zhang, Wei Yang, Fan Wu, Guangrong Zou, Xiaocheng Weng, Xiang Zhou

**Affiliations:** a College of Chemistry and Molecular Sciences , Key Laboratory of Biomedical Polymers of Ministry of Education , The Institute for Advanced Studies , Hubei Province Key Laboratory of Allergy and Immunology , Wuhan University , Wuhan , Hubei 430072 , P. R. China . Email: xzhou@whu.edu.cn ; Email: xcweng@whu.edu.cn ; Fax: +86-27-68756663 ; Tel: +86-27-68756663

## Abstract

5-Formylcytosine (5fC) is known as one of the key players in the process of active DNA demethylation and displays essential epigenetic functions in mammals. In spite of the blooming development of whole genome sequencing methods for this modified cytosine base, the easily operated gene specific-loci detection of 5fC has rarely been reported. Herein, we present a compound-mediated analysis of the content and site of 5fC by the polymerase chain reaction (PCR) assay. The molecule, namely azi-BP, which can selectively label 5fC and form a huge group through a click chemistry reaction, hindering the amplification activity of Taq DNA polymerase, acts as a “roadblock” and enables the quantitative analysis of 5fC by quantitative polymerase chain reaction (qPCR). The existence of 5fC in several fragment-specific genomic DNAs of mouse embryonic stem cells (mESCs) was successfully demonstrated using this method. In addition, the gene fragment containing 5fC can be easily biotinylated and enriched after labeling with azi-BP. Moreover, after azi-BP incorporation, the loss of the exocyclic 4-amino group of 5fC leads to C-to-T conversion and subsequent pairing with adenine (A) in the PCR, which can accurately identify 5fC sites at single-base resolution by site-specific mutation. Azi-BP shows high selectivity to 5fC among all modified pyrimidine bases, revealing that this compound-mediated assay can be applied for content and single-base resolution analysis for gene specific-loci of 5fC.

## Abstract 2

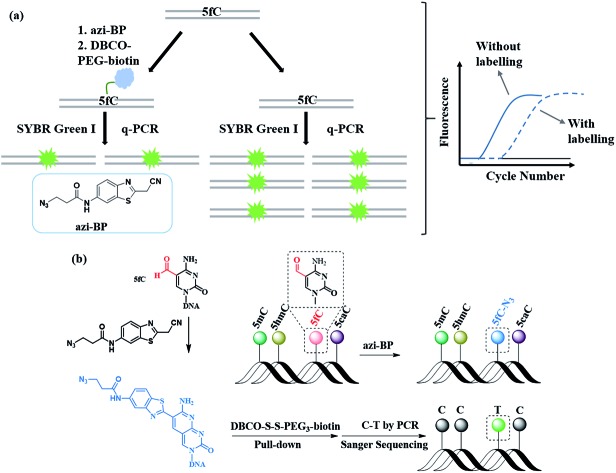
5-Formylcytosine (5fC) is known as one of the key players in the process of active DNA demethylation and displays essential epigenetic functions in mammals.

## Introduction

DNA has been decorated with several modifications in mammalian genomes beyond the four canonical bases.^1^,^2^ 5-Methylcytosine (5mC), deemed as the fifth base in genomic DNA, is an essential epigenetic modification that plays a vital role in gene expression.^3^,^4^ In the process of active DNA demethylation, ten–eleven translocation (TET) proteins are involved in the oxidation of 5mC to 5-hydroxymethylcytosine (5hmC), 5-formylcytosine (5fC) and 5-carboxylcytosine (5caC) in an iterative way.^5^–^7^ 5fC and 5caC can be recognized and excised by mammalian thymine DNA glycosylase (TDG), which uncovers an active DNA demethylation pathway.^8^,^9^ 5fC is found in cells and many organs at a level of 0.02% to 0.002% of cytosine, about 10% to 1% of 5hmC.^10^–^13^ In DNA, 5fC is an essential epigenetic marker and also plays regulatory roles in cellular functions, such as cell differentiation,^14^,^15^ gene regulation^16^ and alterations in DNA structure.^17^–^19^ For example, the formyl group of 5fC increases the acidity, thus weakening the hydrogen bonding and reducing the base pair stability.^19^ Some proteins including transcriptional regulators and chromatin regulators have been found to reveal a strong preference for 5fC.^20^ Recently, it was reported that 5fC can block DNA and RNA polymerases by mediating DNA-histone crosslinking^21^ and contribute to transcriptional regulation and chromatin remodelling.^22^ In view of all factors mentioned above, the detection of 5fC is worthy of attention.

However, it is extremely difficult to detect 5fC because of its low content.^23^ Balasubramanian and co-workers firstly made a breakthrough in enriching 5fC in mESCs using the commercial probe *O*-(biotinylcarbazoylmethyl)hydroxylamine.^24^ To achieve the single-base analysis of 5fC, which is important to provide more information to massively energize the field of epigenetics, He and co-workers developed a fC-Seal method to realize the base-resolution detection of 5fC in genomic DNA using EtONH_2_-modified bisulfite sequencing.^25^ Balasubramanian and co-workers also presented a reduced bisulfite sequencing method (redBS-Seq) through the selective chemical reduction of 5fC to 5hmC, followed by bisulfite treatment.^26^ Next, Yi and co-workers reported two bisulfite-free methods, fC-CET method^27^ and CLEVER-seq,^28^ for the single-base analysis of 5fC. All these excellent studies offer diversified methods to uncover the uncharted territory of 5fC.

Despite the blooming development of whole genome sequencing methods for this modified cytosine base, the easily operated gene specific-loci detection of 5fC is rare, which should be very helpful for further application of 5fC in biological or medical research. For the relatively high reactivity of the formyl group in 5fC, the recognition base on chemical probes is the effective choice on account of its convenience and efficiency. Chemicals containing groups such as amine,^29^ hydroxylamine,^24^,^30^ hydrazine,^31^–^34^ *o*-phenylenediamine^35^,^36^ and indole^37^ were designed as efficient probes for targeting the formyl group. However, a big challenge still remains for the easily operated gene specific-loci detection of 5fC by chemical probes, namely, achieving high selectivity and under mild reaction conditions. In our previous study, we demonstrated that 2-(5-chlorobenzo[*d*]thiazol-2-yl)acetonitrile can selectively tag 5fC to generate a cyclized fluorescent nucleobase (CB-C), firstly realizing both fluorogenic labeling and single-base resolution analysis of 5fC in DNA.^38^ The cyano group of this probe can both react with the 4-amino and 5-formyl groups of 5fC, which highly increase its selectivity compared with other probes targeting only the formyl group.

**Fig. 1:**
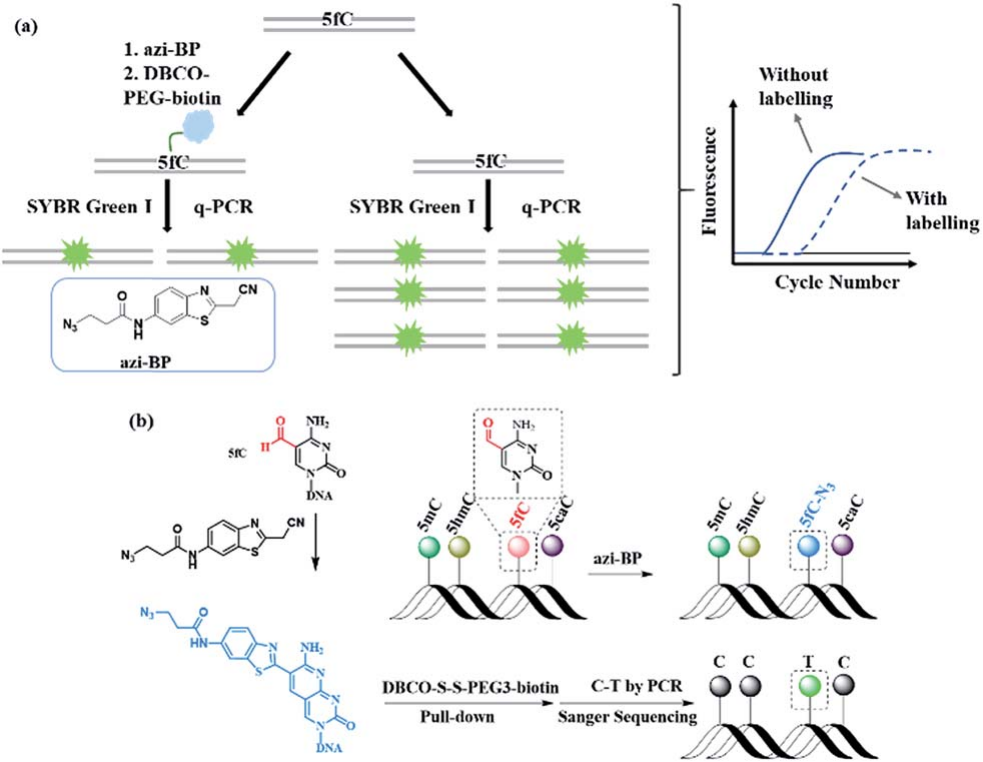
(a) Illustration of the DBCO–azi-BP mediated inhibition of the amplification activity of DNA polymerase. (b) 5fC sites in the tested DNAs undergo azi-BP mediated cyclization labeling and C-to-T transitions.

In this study, we created a new multifunctional molecule, namely azi-BP, to perform content analysis and single-base resolution detection of 5fC at fragment-specific or gene-specific DNA (Fig. 1) based on our previous work.^38^ After labeling with 5fC, the azide group of azi-BP–fC can react with DBCO–biotin reagents through a copper-free click reaction^39^,^40^ and the coupling product with a huge group may act as a “roadblock”, resulting in specific hampering of DNA polymerases on the chemically labeled 5fC in the template DNA, which can be used for 5fC content analysis by qPCR. Moreover, the biotinylation of this probe provides the advantages of efficient enrichment to amplify 5fC in spite of its low abundance. Besides, after being labeled, 5fC loses its exocyclic 4-amino group which is a competent proton donor for canonical base pairing with guanine (G), leading to misreading of 5fC as T during PCR amplification for realizing the single-base resolution analysis of 5fC. Thus, the design can easily identify 5fC within specific loci in an accurate qualitative and quantitative way.

## Results and discussion

### Evaluating the reactivity of azi-BP with ODN-5fC

First, azi-BP was synthesized in four simple steps (Fig. S1–S3†). To explore the feasibility of labeling 5fC with azi-BP, a 9-mer single stranded ODN (ODN-5fC) with one 5fC site was chosen for the preliminary test. The reaction was carried out in HEPES buffer (pH 7.4) at 56 °C for 18 h, followed by RP-HPLC to monitor the reaction. A new peak appeared at 29.2 min (Fig. S4†), indicating that ODN-5fC generated a new product after being labeled with azi-BP. To further confirm the successful labeling of DNA, the purified product was subjected to characterization by MALDI-TOF MS (Fig. S5b†) and enzymatically digested to constitute nucleosides for LC-MS analysis (Fig. S6†). Furthermore, the biotinylated product between azi-BP–fC and DBCO–PEG4–biotin or DBCO–S–S–PEG3–biotin was also verified by HPLC (Fig. S4†) and MADLI-TOF MS (Fig. S5c and d†). We further identified the selectivity of 5fC among all the modified cytosines (5mC, 5hmC, 5fC, 5caC). Polyacrylamide gel electrophoresis (PAGE) analysis confirmed the successful selective labeling of azi-BP into ODN-5fC (Fig. S7a and b†). All these data suggested that ODN-5fC could be labeled with the designed reagent azi-BP and easily biotinylated.

### Reactivity inhibition of DNA polymerase on replication by DBCO–PEG4–biotin

**Fig. 2:**
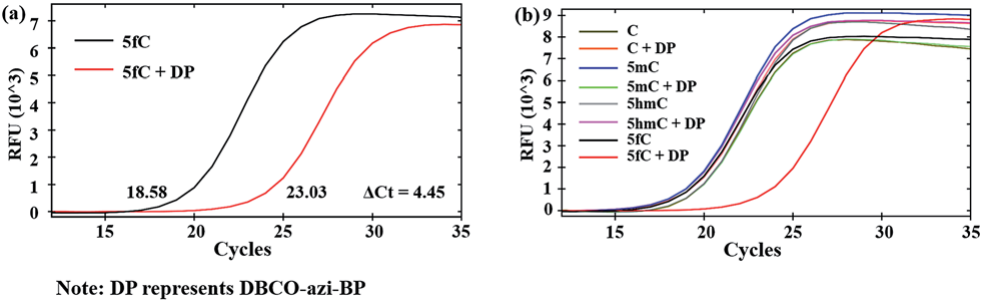
DBCO–azi-BP specifically inhibits the amplification activity of Taq DNA polymerase on 5fC-containing DNA. (a) The qPCR curves of ds ODN–fC in the presence (red line) or absence (black line) of DBCO–azi-BP. (b) The qPCR curves of ds ODNs containing a C, 5mC, 5hmC or 5fC in the presence or absence of DBCO–azi-BP.

Next, 80 bp double-stranded DNA (dsDNA) containing 5fC (80 bp ds-ODN–fC, ESI Table S1†) was chosen for further study and labeled using the protocol described in the ESI.† With the addition of azi-BP and biotinylation by DBCO–PEG4–biotin, we estimated that a much bigger group was formed compared with the original formyl-group, which may create an obstacle for normal enzymatic reactions such as PCR. The most common polymerase, Taq DNA polymerase was selected to test the effect of this huge additional group on the replication of 80 bp ds-ODN–fC. Quantitative-PCR (qPCR) was used to evaluate the replication activity. Cycle of threshold (*C*_t_) is the number of cycles equal to the defined fluorescence signal. A larger numeric value of *C*_t_ indicates a lower amount of amplifiable DNA template, which means that the replication process was disturbed. As expected, after being labeled with DBCO–PEG4–biotin, the *C*_t_ value of 80 bp ds-ODN–fC increased to 23.03 in comparison with unlabeled DNA (18.58) (Fig. 2a). The effect of replication interference was determined from Δ*C*_t_, which can be calculated using the equation: Δ*C*_t_ = *C*_t_ (labeled with the reagent) – *C*_t_ (without labeling). This result demonstrated that the additional group resulting from azi-BP and DBCO–PEG4–biotin (DBCO–azi-BP) could act as a “roadblock” to impede the replication of the 5fC-containing template by Taq DNA polymerase. By contrast, intact 80 bp ds-ODN–fC displayed the same qPCR amplification curve as 80 bp ds ODN–C, and other DNA epigenetic forms 80 bp ds-ODN–mC and 80 bp ds-ODN–hmC (Fig. 2b). What's more, DBCO–PEG4–biotin cannot change the *C*_t_ value of 80 bp ds ODN–C, 80 bp ds ODN–mC and 80 bp ds ODN–hmC, which also underwent the same reaction with DBCO–azi-BP, suggesting the high selectivity of DBCO–azi-BP towards the labeling of 5fC. These results manifest that our design can provide a new approach for detection of 5fC and encourage us to perform further detailed study.

### Linear relationship between the number of 5fC and inhibition efficiency

**Fig. 3:**
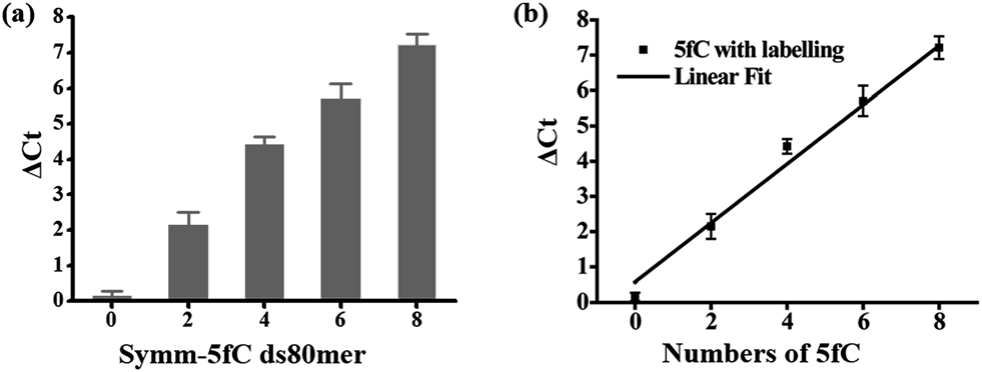
Linear relationship between the number of 5fC and inhibition efficiency by chemical reagent-mediated PCR. (a) Δ*C*_t_ values of ds DNA templates containing different numbers of 5fC (0, 2, 4, 6, 8). (b) Linear relationship between the Δ*C*_t_ value and the number of 5fC in the templates.

Then we investigated whether there is a relationship between the number of 5fC and the inhibition effect on DNA Taq polymerase. To this end, four 80 bp ds-DNAs containing two, four, six and eight 5fC sites (ds-ODN–fC-2, ds-ODN–fC-4, ds-ODN–fC-6, ds-ODN–fC-8, ESI Table S1†) were synthesized as described in ESI.† All these DNAs were similarly labeled with DBCO–azi-BP and used for qPCR analysis. The Δ*C*_t_ value increased from 2.14 to 7.21 with increasing number of 5fC (Fig. 3a). A linear correlation was observed between the Δ*C*_t_ values and the number of 5fC (Δ*C*_t_ = 0.84*X* + 0.57, *X* represents the number of 5fC, *R*^2^ = 0.99; Fig. 3b). This finding apparently shows that the hindering efficiency is positively correlated with the 5fC content within the tested fragments. 5fC may exist under semi-modified conditions in the genome. We tested the mixed dsDNA templates (80 bp ds ODN-C-2 and seq-1 ds-ODN–fC) with varying molar ratios (10 : 0, 8 : 2, 6 : 4, 4 : 6, 2 : 8, 0 : 10) of fC/C at a single specific site. A linear correlation was observed between the Δ*C*_t_ values and the modification ratio (Δ*C*_t_ = 0.77*X* + 0.67, *X* represents lg[5fC/C], *R*^2^ = 0.98; Fig. S8†). These results indicate that our design can detect low ratio modification of 5fC in DNA.

### Evaluating the specificity towards 5fC by mixing with other DNAs

**Fig. 4:**
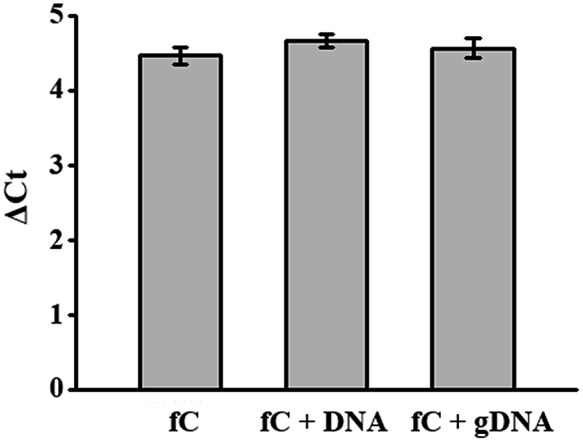
The effect of co-existing DNA for the qPCR assay. The co-existing DNA represents non-fC DNA or genomic DNA of MCF-7.

To investigate whether this detection method still works in a complex environment, 5fC-containing DNA (E-DNA–fC, ESI Table S1†) was mixed with other dsDNA (mixed ds ODN, ESI Table S1†) without the modified site at a molar ratio of 1 : 1. The total amount of dsDNA as the template for qPCR was kept at about 2 pg. The inhibition of the amplification efficiency of labeled fC by DBCO–azi-BP was similar to that of pure fC-containing DNA (Fig. 4). When the ds-DNAs containing fC were further mixed with genomic DNA from MCF-7 cells, there was still no obvious change in the inhibition of DNA Taq polymerase activity caused by labeled fC (Fig. 4). These results suggest that the presence of interfering DNA will not affect the inhibition efficiency of the amplification of ds-DNA–fC caused by DBCO–azi-BP.

### Detection of gene-specific 5fC in genomic DNA by the DBCO–azi-BP mediated qPCR assay

**Fig. 5:**
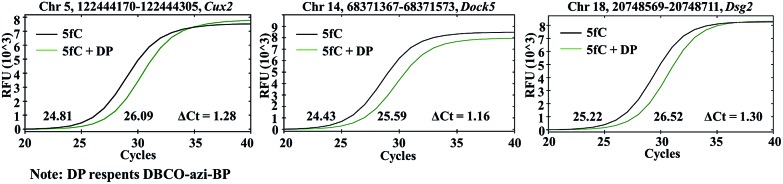
The DBCO–azi-BP mediated qPCR assay for fragment-specific detection of 5fC in genomic DNA of mouse embryonic stem cells (mESCs).

Encouraged by the above results, we aimed at detecting 5fC within some specific genomic DNA regions from mESCs. For this purpose, three specific regions reported to contain 5fC were selected for the test, which are chr5 (122444170–122444305, *Cux2*), chr14 (68371367–68371573, *Dock5*) and chr18 (20748569–20748711, *Dsg2*) respectively (ESI Table S3†).^27^ We used mouse genome (mm9) to identify the regions. As expected, the genomic DNA labeled with DBCO–azi-BP showed a higher *C*_t_ value in qPCR than that in the absence of DBCO–azi-BP (Fig. 5), indicating the presence of 5fC in the tested regions. To validate these 5fC sites in the tested region, Sanger sequencing is made to determine the loci of 5fC in the specific regions (Fig. 7d–f). Collectively, this design will be a potential approach for screening 5fC in the genome.

### Enriching 5fC-containing DNA fragments

**Fig. 6:**
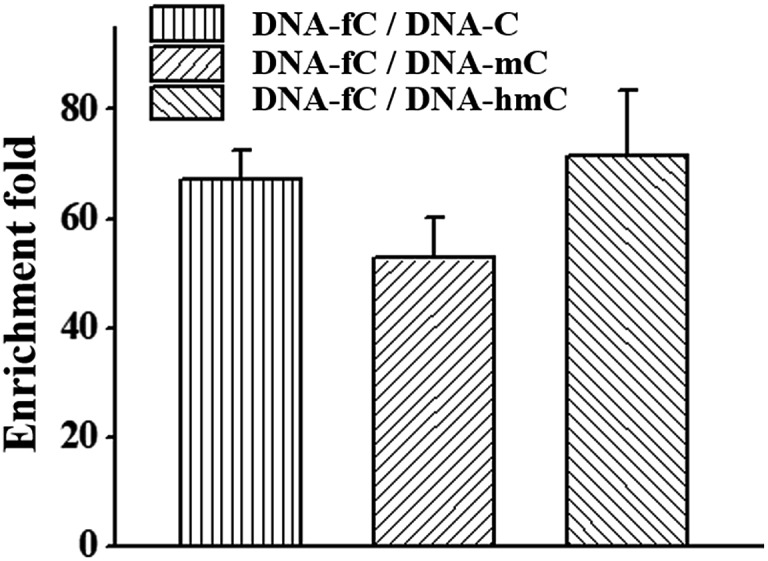
Extent of enrichment of 5fC in double stranded DNA towards C, 5mC and 5hmC ODN sequence.

Besides the advantage of 5fC content analysis, the DBCO–azi-BP can be biotinylated to provide another application to enrich DNA fragments bearing 5fC. To evaluate the enrichment efficiency, four 80 bp dsDNAs (E-DNA–C, E-DNA–mC, E-DNA–hmC, E-DNA–fC) were used as the enrichment models, which had the same sequence except that the 5fC site was replaced by C, 5mC and 5hmC, respectively. These ODNs were subjected to reaction with azi-BP followed by biotinylation. After removing the excess chemical reagent, purified ODNs were obtained for affinity enrichment using streptavidin-coated magnetic beads. The results showed that DNA–fC was enriched over DNA–C, DNA–mC and DNA–hmC by 67 fold, 53 fold and 72 fold respectively (Fig. 6). As mentioned above, when the modification ratio of fC/C is lower, the labeled mixture also has an inhibition effect on DNA polymerase reactivity (Fig. S8†). Combining these results, DBCO–azi-BP had a high selectivity toward 5fC over other modifications and can greatly amplify the signal of 5fC.

### Sanger sequencing for single-base resolution analysis of 5fC

**Fig. 7:**
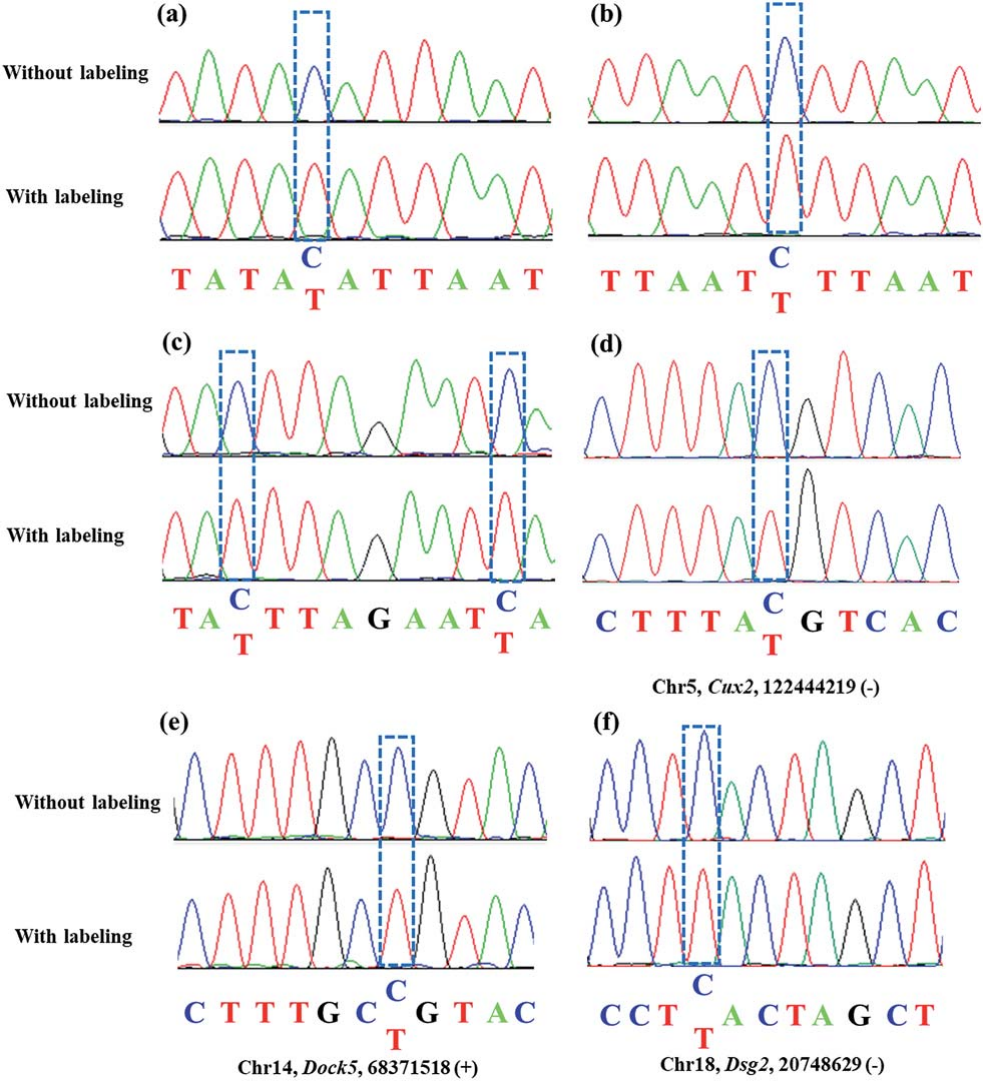
Sanger sequencing analysis of 5fC in the presence of DBCO–azi-BP (bottom) or absence of DBCO–azi-BP (top). (a) One 5fC site of seq-1 ds-ODN–fC. (b) One 5fC site of seq-2 ds-ODN–fC. (c) Two 5fC sites of seq-3 ds-ODN–fC. (d–f) Strand-specific PCR products from mESC gDNA. The base positions originally from 5fC are marked by blue dotted lines.

As mentioned above, azi-BP labeled 5fC generates a cyclic nucleoside, which leads to the loss of the 4-amino group of 5fC and thus fails to pair with G in a canonical base pair way. The loss of the original 4-amino group, which is an efficient proton donor, causes modified 5fC to pair with other bases. An 80 bp-ds DNA containing one 5fC site (seq-1 ds-ODN–fC, ESI Table S1†) was labeled with azi-BP followed by affinity enrichment. The labelled and enriched ODN was amplified by PCR with different polymerases. The PCR products were further applied in TOPO cloning analysis. The Sanger sequencing results showed that the modified 5fC is read as T during the polymerase chain reaction by MightAmp DNA polymerase (Fig. 7a), indicating that the designed reagent can realize a single-base resolution analysis of 5fC. Two more ODNs containing one (seq-2 ds-ODN–fC) and two (seq-3 ds-ODN–fC) 5fC sites were also tested, and the C-to-T conversion in the 5fC site was also observed by Sanger sequencing, which showed that the 5fC sites can be accurately located only by PCR and Sanger sequencing techniques (Fig. 7b and c). Next, the tandem labeling-sequencing analysis was applied to mESC genomic DNA. The gDNA was labelled with chemicals then used for strand-specific PCR and further for TOPO cloning analysis. An obvious C-to-T transition was observed in the tested fragments (Fig. 7d–f and S10†) and the particular sites are in chromosome 15 (*Cux2* gene, 122444219, –), chromosome 14 (*Dock5* gene, 68371518, +), chromosome 18 (*Dsg2*, 20748629, –), chromosome 17 (*Epcam* gene, 88039806, +), and chromosome 17 (*Epcam* gene, 88039703, –), which corresponded to the reported literature.^27^ Combining the enrichment analysis and Sanger sequencing analysis, our design can be used as a potential strategy for single-base resolution analysis of 5fC in genomic DNA.

## Conclusions

Herein, we demonstrated a detection method for 5-formylcytosine by the polymerase chain reaction with incorporation of DBCO–azi-BP, which could achieve quantitative and single-base resolution analysis within gene specific-loci. At first, 5fC-containing DNA was specifically labeled with azi-BP, then the azide group reacted with DBCO–biotin reagents by click chemistry. The selectivity of 5fC labeling was verified by comparison with other control ODNs bearing C, 5mC, and 5hmC which were also subjected to the same reaction conditions. In principle, there are two advantages of labeling and biotinylation using our design reagent. First, it increases the size of 5fC and further blocks the replication activity of taq DNA polymerase. During the process of polymerase chain reaction, the labeled 5fC in the template for PCR will hamper the replication activity and the effect can be quantified by qPCR. By qPCR analysis, the ODN containing 5fC revealed an obvious change of *C*_t_ value, indicating that the numeric value of *C*_t_ can be used to evaluate the existence of 5fC. What‘s more, with increasing number of 5fC in ODNs, the *C*_t_ values also increased with excellent linear correlation, suggesting the feasibility of quantitative determination of 5fC. While co-existing with other DNAs, the inhibition of Taq DNA polymerase by DBCO–azi-BP was not disturbed and remained specific for 5fC-containing DNA. Three specific fragments of mESCs reported to contain 5fC were successfully measured by our strategy, indicating that our approach serves as an efficient way for convenient detection of 5fC in genomic DNA.

The second advantage of our method is that the specific 5fC-containing gene segment can be enriched and exhibits single-base resolution analysis for the site mutation of 5fC during PCR. The labeled 5fC bearing a biotin group allows us to selectively pull down the fC-containing DNA fragment. Because of the cyclization reaction between 5fC and azi-BP, the 4-amino group of 5fC is no longer a proton donor and thus may fail to pair with G. By Sanger sequencing, we find that the labeled 5fC is read as T during the polymerase chain reaction. The selective enrichment of 5fC and the C-to-T transition during PCR may help realize the single-base resolution analysis of 5fC across the genome.

In summary, the current study has developed a chemical approach for epigenetic modification detection using a selective 5fC labeling reagent. The PCR process was obstructed and the canonical base-pairing was disturbed by the labeled 5fC, which helps realize both content detection and single-base resolution analysis of 5fC. The specific gene segment with 5fC can be enriched to reduce the background noise after reagent labeling. To our knowledge, this is the first design of a multifunctional chemical reagent for analysis of 5fC. In general, our finding can serve as a potential method to provide high throughput analysis of 5fC across the genome for biological and medical studies and the principle of this method might be used for the detection of other DNA epigenetic modifications.

## Conflicts of interest

There are no conflicts to declare.

## Supplementary Material

Supplementary information
