# Supplementary material for: Gene specific-loci quantitative and single-base resolution analysis of 5-formylcytosine by compound-mediated polymerase chain reaction
Source: Chem Sci. 2018 Mar 19;9(15):3723–8. doi: 10.1039/c8sc00493e (PMC5939610; doi:10.1039/c8sc00493e)
Supplement: Supplementary file 1 [file SC-009-C8SC00493E-s001.pdf]

Electronic Supplementary Material (ESI) for Chemical Science.
This journal is © The Royal Society of Chemistry 2018

Supporting Information

Gene specific-loci quantitative and single-base resolution analysis of 5-formylcytosine by compound-mediated polymerase chain reaction

Yafen Wang[1,†], Chaoxing Liu[1,†], Xiong Zhang[1,†], Wei Yang[1], Fan Wu[1], Guangrong Zou[1], Xiaocheng Weng[1,*] and Xiang Zhou[1,*]

[1]College of Chemistry and Molecular Sciences, Key Laboratory of Biomedical Polymers of Ministry of Education, The Institute for Advanced Studies, Hubei Province Key Laboratory of Allergy and Immunology, Wuhan University, Wuhan, Hubei, 430072, P. R. China.

[*]Corresponding Author, E-mail: xcweng@whu.edu.cn; xzhou@whu.edu.cn; Fax: +86-27-68756663; Tel: +86-27-68756663

[†]These authors contributed equally.

List of Contents:

1	Materials and methods	
2	Synthesis	Figure S1-S3
3	HPLC data	Figure S4
4	DNA MALDI-TOF Mass Spectra	Figure S5
5	LC-MS of digesting DNA	Figure S6
6	Polyacrylamide gel electrophoresis analysis	Figure S7
7	Quantitative evaluation of modification ratio by Biotin-azi-BP-mediated PCR	Figure S8
8	Calibration line of qPCR for enrichment analysis	Figure S9
9	Sanger sequencing analysis of 5fC in genomic DNA of mESCs	Figure S10
10	The sequences used for this study	Table S1
11	The template used for synthesis of ds DNA	Table S2
12	The sequences of primers used in this study	Table S3

1. Materials and methods

Materials and chemicals. All chemicals were purchased from Beijing Innochem Sci. & Tech. Co. Ltd. (Beijing, China) unless mentioned otherwise. All of the unmodified oligonucleotides were synthesized and purified by GeneCreate Co., Ltd. (Wuhan, China). And the oligonucleotides containing 5-formylcytosine (ODN-5fC), Hot Start Taq polymerase, MightyAmp DNA polymerase and dNTP (N = A, T, C, G) were purchased from Takara Biotechnology Co., Ltd. (Dalian, China). Dream Taq polymerase was brought from ThermoFisher Scientific (USA). 2× Hieff™ PCR SYBR® Green Master Mix were ordered from YEASEN (Shanghai, China). dmCTP was purchased from New England BioLabs (USA). dhmCTP and dfCTP were obtained from TriLink Biotechnologies (USA). DNA Clean & Concentrator™-5 kit and Degradase Plus were purchased from Zymo Research (USA). Mini quick spin oligo columns were ordered from Roche (Germany). pClone007 Simple Vector Kit was purchased from TSINGKE Biological Technology (Beijing, China). The mESCs culture medium was purchased from Cyagen Biosciences (China). Amicon Ultra-0.5 mL Centrifugal Filters 10K were obtained from Millipore (USA). The nucleic acid stains Super GelRed (NO.: S-2001) was bought from US Everbright Inc. (Suzhou, China).

Synthesis of 80 bp ds ODN-C, ODN-mC, ODN-hmC, ODN-fC by polymerase chain reaction. To synthesize 5mC/5hmC/5fC containing oligodeoxynucleotide, modified nucleotides were incorporated into PCR. 80 bp ds ODN-C, 80 bp ds ODN-mC, 80 bp ds ODN-hmC and 80 bp ds ODN-fC (See Table S1) were synthesized using template 1, forward primer 1 and reverse primer 1 in the presence of dATP, dTTP, dGTP and dXTP (for 80 bp ds ODN-C, X = C, for 80 bp ds ODN-mC, X = 5mC, for 80 bp ds ODN-hmC, X = 5hmC and for 80 bp ds ODN-fC, X = fC). PCR reactions were prepared with 10 × DreamTaq Buffer (5 μL), 2 μL forward primer (10 μM), 2 μL reverse primer (10 μM), 1 μL template (100 nM), 1 μL dATP (10 mM), 1 μL dTTP (10 mM), 1 μL dGTP (10 mM), 1 μL dXTP (10 mM), 5 U DreamTaq Polymerase and ddH_2O to give a final volume of 50 μL. The mixture was then subjected to the following thermal cycle: 95 °C for 3 min, 35 cycles of (95 °C for 10 s, 60 °C for 30 s, 72 °C for 90 s), 72 °C for 3 min using a T100 Thermal Cycler (BioRad). For synthesis of 80 bp ds ODN-mC, the mixture components were changed with minor modifications. PCR reactions were prepared with 10 × Hot Start Taq Buffer (5 μL), 2 μL forward primer (10 μM), 2 μL reverse primer (10 μM), 1 μL template (100 nM), 1 μL dATP (10 mM), 1 μL dTTP (10 mM), 1 μL dGTP (10 mM), 1 μL dmCTP (10 mM), 2.5 U Hot Start Taq Polymerase and ddH_2O to give a final volume of 50 μL. The mixture was then subjected to the following thermal cycle: 95 °C for 15 min, 35 cycles of (94 °C for 30 s, 60 °C for 30 s, 72 °C for 30 s), 72 °C for 3 min using a T100 Thermal Cycler (BioRad). The PCR product was purified using a DNA Clean & Concentrator™-5 kit according to the manufacturer's instructions. The PCR product was confirmed by 20% native polyacrylamide gel electrophoresis (PAGE) analysis. DNA concentration was quantified by NanoDrop 2000c (Thermo Scientific, USA).

Synthesis of 80 bp dsDNA containing different numbers of fC. To get 80 bp dsDNA containing different numbers of 5fC, Template 2-5 were used as the templates for synthesis of 80 bp ds DNA containing different numbers of 5fC respectively. The protocol was the same as described above.

Chemical labeling reaction with fC-ODN. For the reaction between ODNs and azi-BP, 1 μL ODN (100 μM) was added into the mixture of 5 μL HEPES buffer (1 M, pH 7.4) and 19 μL ddH_2O and

25 μL azi-BP (100 mM, dissolved in DMSO). After vortexing and centrifugation, the mixture was incubated in a thermos-shaker (Ningbo Biocotek Scientific Instrument Co., Ltd., China, 1500 r.p.m.) at 56 ℃ for 18 h. The product was purified by HPLC. The purified product was incubated with 10 μL DBCO-PEG4-biotin (10mM) or DBCO-S-S-PEG_3-biotin (20 mM) at 37 ℃ for 2 h. After that, the mixture was purified by HPLC.

For the reaction between 80 bp ds ODN and azi-BP, 1 μg DNA was used as input, and the condition of the reaction was the same as the protocol described above. The minor difference was that the excess reagent was removed by DNA Clean & Concentrator^{TM}-5 kit according to the manufacturer's instructions.

HPLC analysis of ODNs. ODNs were treated with azi-BP or further treated with DBCO-PEG4-biotin or DBCO-S-S-PEG_3-biotin through the above protocol, then HPLC data analysis was performed on LC-6AD (Shimadzu, Japan) which was equipped with an Inertsil ODS-SP column (5 μm, 250×4.6 mm) (GL Science lnc. Japan) with mobile phase A (CH_3CN) and B (100 mM TEAA buffer, pH=7.0) at a flow rate of 1 mL/min at 35°C (A Conc.: 5-5-30-100% / 0-5-30-40 min).

Enzymatic digestion of ODNs. Purified DNA in the presence of 10 × Degradase Plus reaction buffer (2.5 μL) and Degradase Plus (1 μL) in a final volume of 25 μL was digested to its corresponding nucleosides by incubation at 37 °C for 2 h. The solution mixture was purified by an ultrafiltration tube (10 kDa cutoff, Amicon, Millipore) to remove the enzymes.

LC-MS analysis of chemical labeling fC-ODN. LC-MS was performed on a Thermo Scientific Dionex Ultimate 3000 hybrid LTQ Orbitrap Elite Velos Pro system, using a Shimadzu Inertsil ODS-3 C18 column (5 μM, 2.1 x 250 mm). The column temperature was set at 35 °C. Water containing 0.1% formic acid (v/v, solvent A) and methanol with 0.1% formic acid (v/v, solvent B) were employed as mobile phase with a flow rate of 0.2 mL/min. A gradient for LC-MS was set as follows: 5% B to 80% B over 20 min, 80% B for 7 min, 80% to 5% B over 3 min, 5% B for 5 min. Identity of product was confirmed by ESI-MS/MS.

qPCR analysis of different modification ratio of fC/C. seq-1 ds-ODN-fC and 80 bp ds ODN-C-2 were mixed with different molar ratio (0:10, 2:8, 4:6, 6:4, 8:2, 10:0). The mixed DNA was labeled with azi-BP and then incubated with DBCO-PEG4-biotin. The purified product were further for qPCR analysis.

The protocol of qPCR analysis of 5fC. qPCR was performed using a CFX-96 Real-Time System (Bio-Rad, USA). The mixture contained 1 μL template of the labeled DNA or unlabeled DAN, 1 μL forward primer (10 μM), 1 μL reverse primer (10 μM), 10 μL 2 × Hieff^{TM} PCR SYBR$^{®}$ Green Master Mix and 7 μL ddH_2O to give a final volume of 20 μL. The mixture was subject to qPCR according to the following thermal cycle: 95 °C for 5 min, 40 cycles of (95 °C for 10 s, 60 °C for 30 s, 72 °C for 20 s).

Chemical labeling-mediated qPCR assay. 1 pg or other concentration of labeled 80 bp DNA was used as template for qPCR assay. The corresponding sequence without treatment was used as control. The Ct values corresponding to labeled fC and unlabeled fC are named as Ct_l and Ct_0,

respectively. $\Delta Ct = Ct_1 - Ct_0$.

Sanger sequencing and TOPO cloning analysis after labeling of 5fC-containing dsDNAs. Chemically labeled 5fC-dsDNAs were prepared as described above and used as templates for PCR amplification. And the chemically labeled 5fC-dsDNAs were used as the template for PCR amplification. MightyAmp DNA polymerase was used for amplification. The PCR was performed according to the following thermal cycle: 98 °C for 2 min, 35 cycles of (98 °C for 10 s, 60 °C for 15 s, 68 °C for 30 s), 68 °C for 2 min. PCR product was purified by DNA Clean & Concentrator™-5 kit and the concentration was quantified by NanoDrop 2000c. The fragment was inserted into a plasmid T-vector (pClone007 Simple Vector Kit) according to the manufactuer's instructions for Sanger sequencing.

DNA enrichment protocol. The enrichment procedure was used according to the manufacturer's instructions with some modifications to the use of Dynabeads™ MyOne™ Streptavidin C1 (ThermoFisher Scientific). Briefly, 1× binding and washing (B&W) buffer (pH 7.5) was added with 0.05% Tween-20. Beads (20 μL) were washed with 200 μL 1 × B&W buffer for three times and then resuspended in 20 μL 2 × B&W buffer. Input DNA (1 ng) and calf thymus (10 μg, sonicated into 100-200 bp, Sigama) were mixed and made up to a final volume of 20 μL and then added to the magnetic beads. Then pipette the entire system up and down to mix thoroughly. The mixture was incubated at room temperature for 15 minutes on a nutator. After that, the tube was placed on a magnet for 5 minutes before discarding the supernatant, and beads were washed with 500 μL 1 × B&W buffer for three times. Then 100 μL freshly prepared DTT (50 mM) was added to the beads and the mixture was incubated at 37 °C for 2 h. The supernatant containing the desired DNA was purified with DNA Clean & Concentrator™-5 kit to remove DTT.

qPCR analysis for chemical enrichment studies. qPCR was performed using a CFX-96 Real-Time System (Bio-Rad, USA). The mixture contained 1 μL template, 1 μL forward primer (10 μM), 1 μL reverse primer (10 μM), 10 μL 2 × Hieff™ PCR SYBR® Green Master Mix and ddH_2O to give a final volume of 20 μL. The mixture was subject to qPCR according to the following thermal cycle: 95 °C for 5 min, 40 cycles of (95 °C for 10 s, 60 °C for 30 s, 72 °C for 20 s). DNA concentration was quantified by comparison with calibration lines of known concentration of input ODNs.

Mouse embryonic stem cells culture and DNA extraction. Mouse embryonic stem cells (mESCs) were cultured in Dulbecco's modified Eagle's medium (DMEM) with 15% fetal bovine serum (FBS), penicillin/streptomycin, non-essential amino acids, L-glutamate, beta-Mercaptoethanol, leukemia inhibitory factor (LIF), 2I-004 and 2I-006. mESCs were plated in culture dishes pretreated with 0.1% gelatin then incubated in humidified 37 °C incubator supplied with 5% CO_2. Genomic DNA was extracted from the harvested cells using a DNeasy® Blood & Tissue Kit (Qiagen, Germany) following the manufacturer's instructions.

2. Synthesis

2-amino-5-nitrobenzenethiol was synthesized according to the previous report.[1] 2-amino-5-nitrobenzenethiol (3.40 g, 20 mmol, 1.0 eq) and malononitrile (1.32 g, 20 mmol, 1.0 eq) were dissolved into 50 mL ethanol at a 100 mL round bottom flask . Then, 5 mL acetic acid was added

while the reaction mixture was kept stirring. After 12 h stirring at 60°C, the orange precipitate was isolated by filtration, and purified through column chromatography using eluent PE / EtOAc (2 : 1) to yield 2 g (50%) as a orange solid. ^{1}H NMR (300 MHz, DMSO) δ = 9.21 (d, *J*=2.1, 1H), 8.36 (dd, *J*=9.0, 2.2, 1H), 8.23 (d, *J*=8.9, 1H), 4.86 (s, 2H). ^{13}C NMR (75 MHz, DMSO) δ = 168.16, 156.30, 145.08, 136.27, 123.57, 122.17, 120.00, 116.56, 23.28. HRMS $C_9H_6N_3O_2S^+$ $[M+H]^+$ calculated 220.01752, found 220.01807.

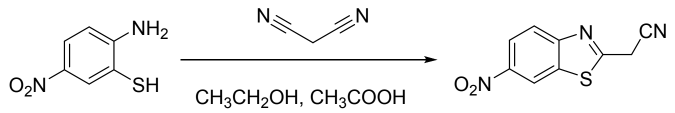

Figure S1. Synthesis of 2-(6-nitrobenzo[d]thiazol-2-yl)acetonitrile.

2-(6-nitrobenzo[d]thiazol-2-yl)acetonitrile (219 mg, 1mmol, 1.0 eq) and ammonium chloride (535 mg, 10 mmol, 10.0 eq) were dissolved into 40 mL methanol in 100 mL round bottom flask. After 5 minutes stirring at room temperature, zinc powder (1.3 g, 20 mmol, 20.0 eq) was added. Then, the reaction mixture was still kept stirring at room temperature for 30 minutes and the zinc powder was filtered off through adding the diatomaceous earth filter. Subsequently, the collected reaction solution was dried in vacuo. The product was purified by column chromatography using eluent PE / EtOAc (2 : 1) to yield 100 mg (53%) as a orange solid. ^{1}H NMR (300 MHz, DMSO) δ =7.57 (d, *J*=9, 1H), 6.99 (d, *J*=1.8, 1H), 6.70 (dd, *J*=8.7, 1.8, 1H), 5.39(s, 2H), 4.49 (s, 2H). ^{13}C NMR (101 MHz, DMSO) δ 153.53, 147.91, 144.20, 137.32, 123.30, 117.38, 115.40, 104.20, 22.37. HRMS $C_9H_8N_3S^+$ $[M+H]^+$ calculated 190.04334, found 190.04252.

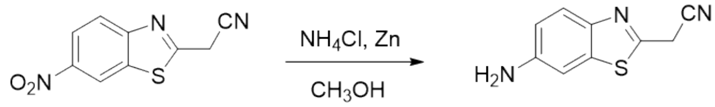

Figure S2. Synthesis of 2-(6-aminobenzo[d]thiazol-2-yl)acetonitrile.

3-azidopropionic was synthesized according to the previous report.[2] 3-azidopropionic (230 mg, 2 mmol) was dissolved in anhydrous dichloromethane (40 mL), and then the thionyl chloride (1 mL, 5 mmol) was added dropwise. The reaction mixture was refluxed for 2 hours at 40°C after which dichloromethane and remaining thionyl chloride were removed in vacuo. Subsequently, the synthesized 3-azidopropionyl Chloride was dissolved in anhydrous trichloromethane (8 mL), and treated with a solution of 2-(6-aminobenzo[d]thiazol-2-yl)acetonitrile (30 mg, 0.16 mmol) in anhydrous trichloromethane (2 mL). The reaction mixture was kept stirring at 40°C for 30 minutes. The product was purified by silica gel column chromatography using eluent CH_2Cl_2 / MeOH (200 : 1) to yield 20 mg (45%) as a light orange powder. ^{1}H NMR (400 MHz, DMSO-d_6) δ 10.38 (s, 1H), 8.53 (d, J = 2.0 Hz, 1H), 7.97 (d, J = 8.8 Hz, 1H), 7.56 (dd, J = 8.8, 2.1 Hz, 1H), 4.71 (s, 2H), 3.64 (t, J = 6.2 Hz, 2H), 2.67 (t, J = 6.3 Hz, 2H). ^{13}C NMR (101 MHz, DMSO-d_6) δ 169.43, 159.57, 148.73, 137.23, 136.31, 123.17, 119.31, 117.13, 112.01, 47.26, 36.10, 22.74. HRMS $C_{12}H_{11}N_6OS^+$ $[M+H]^+$ calculated 287.07096, found 287.07049.

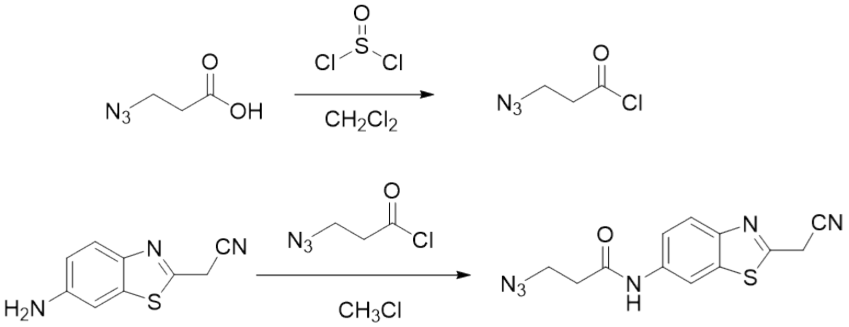

Figure S3. Synthesis of 3-azido-N-(2-(cyanomethyl)benzo[d]thiazol-6-yl)propanamide (azi-BP).

3. HPLC data

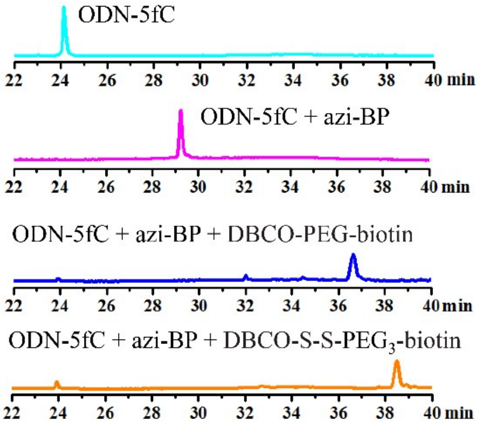

Figure S4. HPLC detection of ODN. (first line) ODN-5fC, (second line) ODN-5fC after reaction with azi-BP, (third line) ODN-5fC after reaction with azi-BP, then linked with DBCO-PEG-biotin, (fourth line) ODN-5fC after reaction with azi-BP, then linked with DBCO-S-S-PEG_3-biotin.

4. DNA MALDI-TOF Mass Spectra

(a) 5'- AGAT5fCGTAT-3'

calculated 2765.5, found 2761.9.

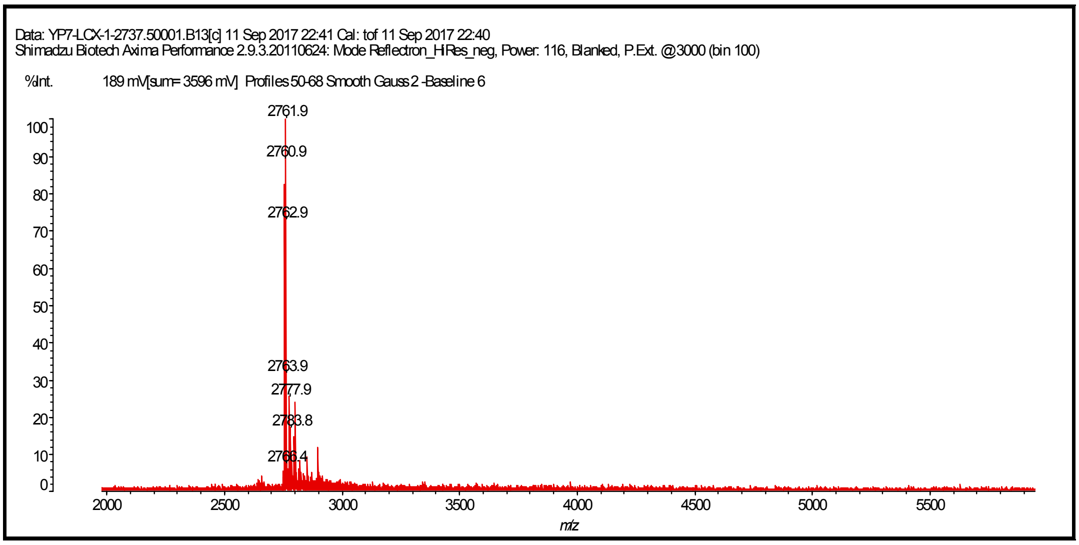

(b) 5'- AGAT5fCGTAT-3' ⟶ 5'- AGAT(azi-BP-5fC)GTAT-3'

calculated 3033.6, found 3033.9.

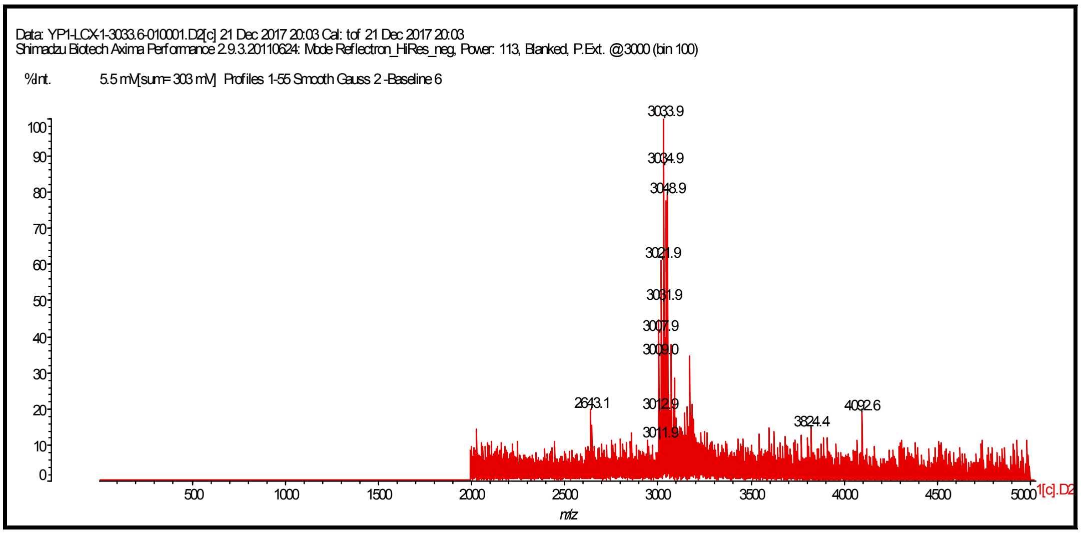

(c) 5’- AGAT(azi-BP-5fC)GTAT-3’ ⟶ 5’- AGAT(azi-BP-5fC+DBCO-PEG-biotin)GTAT-3’

calculated 3783.5, found 3782.7.

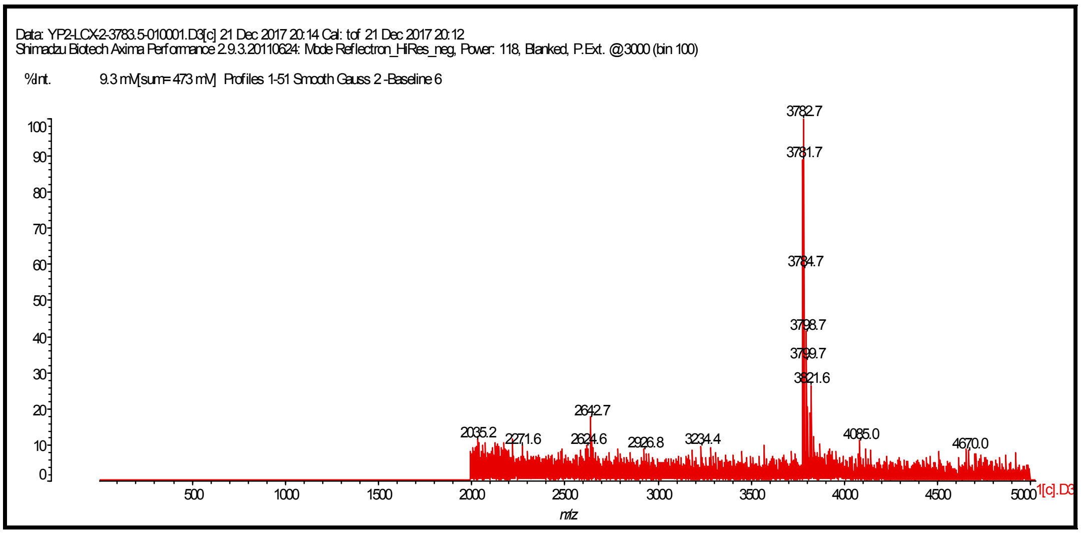

(d) 5’- AGAT(azi-BP-5fC)GTAT-3’ ⟶ 5’- AGAT(azi-BP-5fC+DBCO-S-S-PEG_3-biotin) GTAT-3’

calculated 3901.9, found 3901.3.

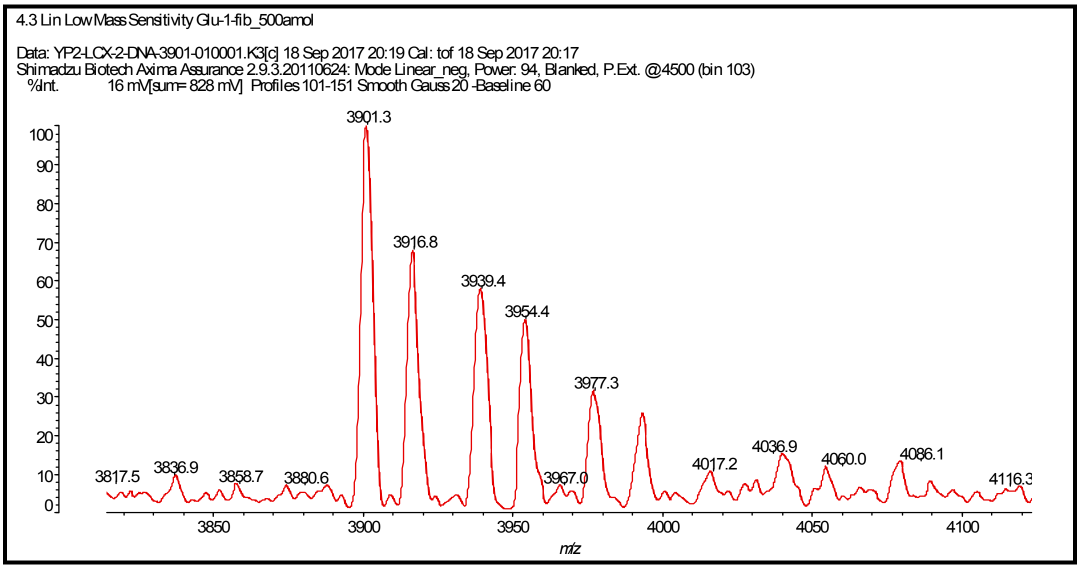

Figure S5. DNA MALDI-TOF Mass Spectra. (a) ODN-5fC, (b) ODN-5fC after reaction with azi-BP, (c) ODN-5fC after reaction with azi-BP, then linked with DBCO-PEG-biotin, (d) ODN-5fC after reaction with azi-BP, then linked with DBCO-S-S-PEG_3-biotin.

5. LC-MS of digesting DNA

(a)

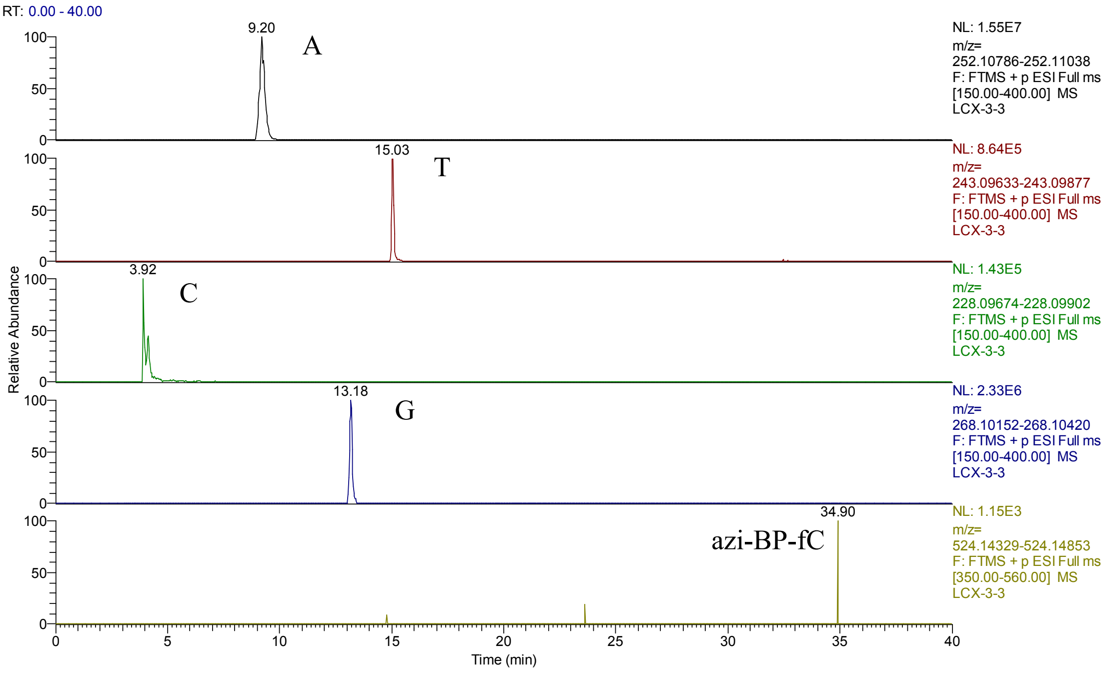

(b)

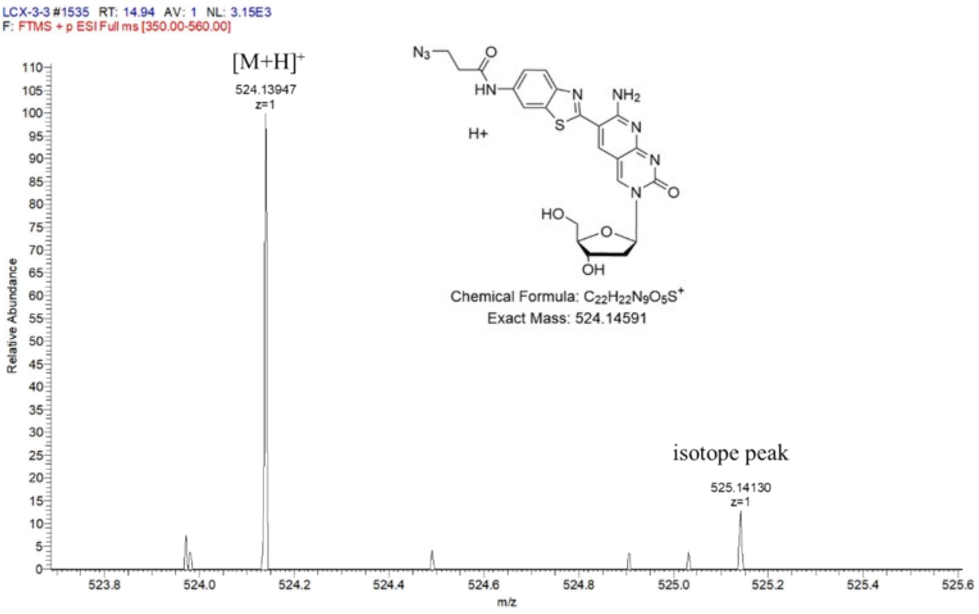

Figure S6. HPLC-MS detection of digestion of ODN-5fC after reaction with azi-BP. (a) HPLC-MS extracted $[M+H]^+$ ion count for A, T, C, G, azi-BP-fC after digestion of DNA from the ODN-5fC after reaction with azi-BP. (b) HRMS (ESI+) of azi-BP-fC in HPLC-MS after digestion, HRMS $C_{22}H_{22}N_9O_5S^+$ $[M+H]^+$ and isotope.

6. Polyacrylamide gel electrophoresis analysis

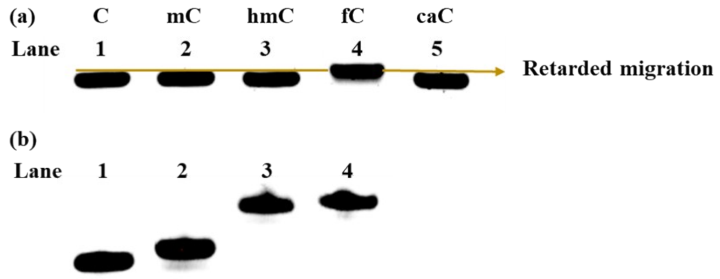

Figure S7. Polyacrylamide gel electrophoresis analysis the labelled DNA. (a) Five different 9 mer ODNs containing C, mC, hmC, fC and caC were incubated with azi-BP. Only ODN bearing 5fC had a retarded migration, indicating that the azi-BP was selectively label into fC. (b) Lane 1: ODN-5fC without treatment; lane 2: ODN-5fC was treated with azi-BP; lane 3: ODN-5fC was treated with azi-BP and DBCO-PEG4-biotin; lane 4: ODN-5fC was treated with azi-BP and DBCO-S-S-PEG$_3$-biotin.

7. Quantitative evaluation of modification ratio by Biotin-azi-BP-mediated PCR

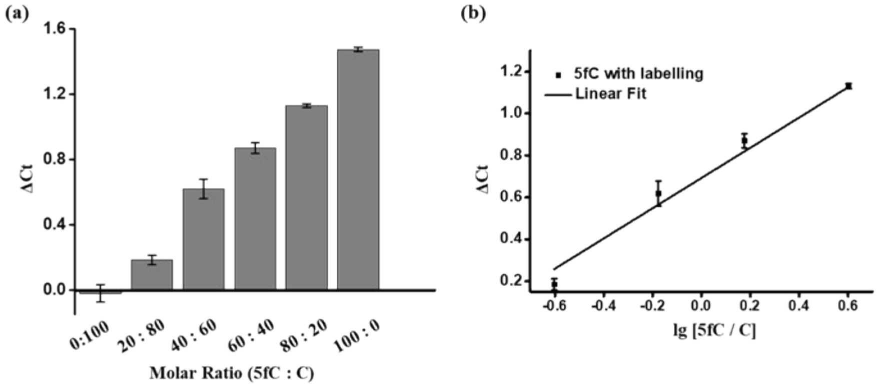

Figure S8. Linear relationship between the modification ratio and inhibition efficiency by chemical reagents-mediated PCR. (a) The ΔCt values of ds DNA templates containing different the modification ratio (0, 20, 40, 60, 80, 100%). (b) The linear relationship between the ΔCt value and the modification ratio.

8. Calibration line of qPCR for enrichment analysis

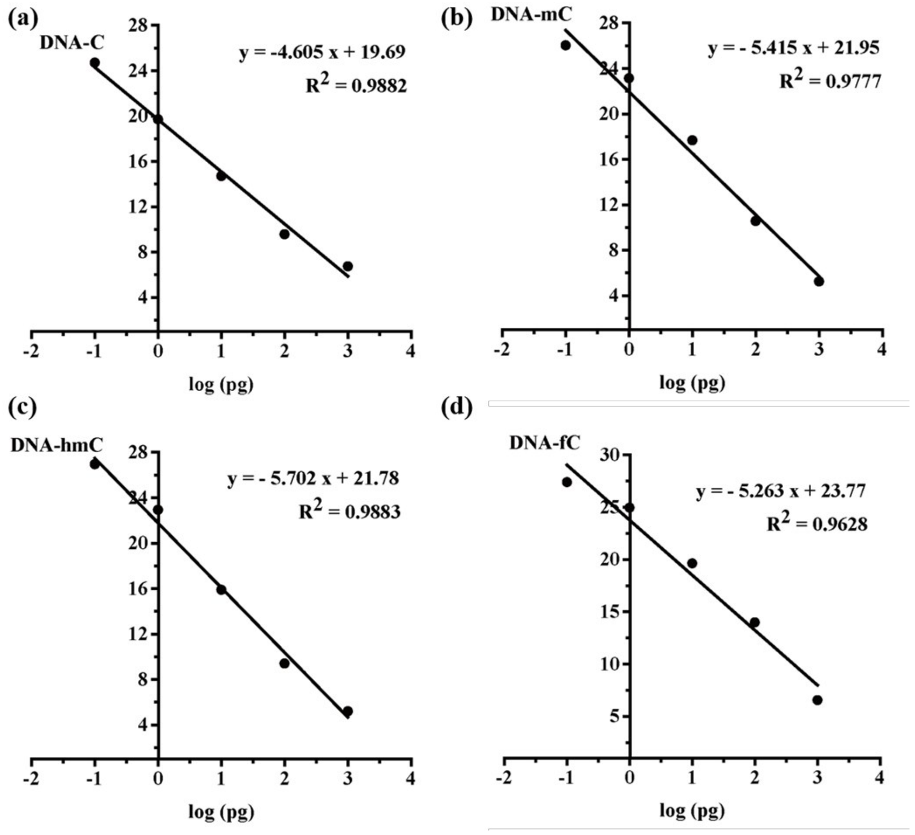

Figure S9. Example calibration line of DNA-C, DNA-mC, DNA-hmC and DNA-fC for enrichment analysis.

9. Sanger sequencing analysis of 5fC in genomic DNA of mESCs.

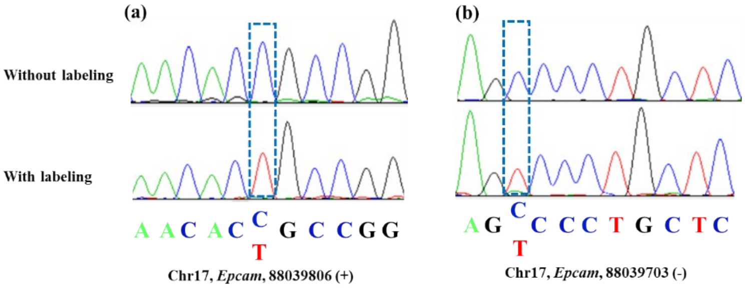

Figure S10. Sanger sequencing analysis of 5fC in the presence of DBCO-azi-BP (bottom) or absence of DBCO-azi-BP (top). (a) and (b) Strand-specific PCR products from mESCs gDNA. The base positions originally from 5fC is surrounded by blue dotted lines.

10. The sequences used in this study

Table S1. The sequences of the oligodeoxynucleotides used in this study.

Note: [a] The corresponding templates were used listed in Table S2. [b] The corresponding primers were used listed shown in Table S3, F is short for forward, R is short for reverse.

ODNs	Sequence
ODN-5fC	AGAT5fCGTAT
ODN-C	AGATCGTAT

ODN-mC	AGATmCGTAT
ODN-hmC	AGAThmCGTAT
ODN-caC	AGATcaCGTAT
80 bp ds<br>ODN-fC<br>(Template 1)[a]<br>F 1, R 1)[b]	5'-<br>TTCTTCTACATCATCTCCCTCTAACCTCCTATATGTA5fCTTAG<br>AAT5fCAATTAGGTAGATTAGAGAGTAGTAGTGGTGAGGA-3'<br>Complementary sequence 3'-<br>AAGAAGATGTAGTAGAGGGAGATTGGAGGATATA5fC ATGAAT<br>5fCTTAGTTAATCCATCTAATCTCTCATCATCACCACTCCT -5'
80 bp ds<br>ODN-C<br>(Template 1)[a]<br>F 1, R 1)[b]	5'-<br>TTCTTCTACATCATCTCCCTCTAACCTCCTATATGTACTTAGAA<br>TCAATTAGGTAGATTAGAGAGTAGTAGTGGTGAGGA-3'<br>Complementary sequence 3'-<br>TTCTTCTACATCATCTCCCTCTAACCTCCTATATGTACTTAGAAT<br>CAATTAGGTAGATTAGAGAGTAGTAGTGGTGAGGA-5'
80 bp ds<br>ODN-mC<br>(Template 1)[a]<br>F 1, R 1)[b]	5'-<br>TTCTTCTACATCATCTCCCTCTAACCTCCTATATGTA5mCTTAGA<br>AT5mCAATTAGGTAGATTAGAGAGTAGTAGTGGTGAGGA-3'<br>Complementary sequence 3'-<br>AAGAAGATGTAGTAGAGGGAGATTGGAGGATATA5mC ATGAA<br>T5mCTTAGTTAATCCATCTAATCTCTCATCATCACCACTCCT -5'
80 bp ds ODN-<br>hmC<br>(Template 1)[a]<br>F 1, R 1)[b]	5'-<br>TTCTTCTACATCATCTCCCTCTAACCTCCTATATGTA5hmCTTAG<br>AAT5hmCAATTAGGTAGATTAGAGAGTAGTAGTGGTGAGGA-3'<br>Complementary sequence 3'-<br>AAGAAGATGTAGTAGAGGGAGATTGGAGGATATA5hmC<br>ATGAA<br>T5hmCTTAGTTAATCCATCTAATCTCTCATCATCACCACTCCT -5'
ds ODN-fC-2<br>(Template 2)[a]<br>F 1, R 1)[b]	5'-<br>TTCTTCTACATCATCTCCCTCTAACCTCCTATATTA5fCGTT<br>ATAATAAATTAGGTAGATTAGAGAGTAGTAGTGGTGAGGA-3'<br>Complementary sequence 3'-<br>AAGAAGATGTAGTAGAGGGAGATTGGAGGATATAATG5fCAAT<br>ATTATTTAATCCATCTAATCTCTCATCATCACCACTCCT-5'
ds ODN-fC-4<br>(Template 3)[a]<br>F 1, R 1)[b]	5'-<br>TTCTTCTACATCATCTCCCTCTAACCTCCTATATTA5fCGTTA<br>5fCGATAAATTAGGTAGATTAGAGAGTAGTAGTGGTGAGGA-3'<br>Complementary sequence 3'-<br>AAGAAGATGTAGTAGAGGGAGATTGGAGGATATAATG5fCAA<br>TG5fCTATTTAATCCATCTAATCTCTCATCATCACCACTCCT -5'
ds ODN-fC-6<br>(Template 4)[a]<br>F 1, R 1)[b]	5'-<br>TTCTTCTACATCATCTCCCTCTAACCTCCTATATTA5fCGTTA5fC<br>GATA5fCGTTAGGTAGATTAGAGAGTAGTAGTGGTGAGGA-3'<br>Complementary sequence 3'-

	AAGAAGATGTAGTAGAGGGAGATTGGAGGATATAATG5fCAAT G5fC TATG5fC AATCCATCTAATCTCTCATCATCACCACTCCT -5’
ds ODN-fC-8 (Template 5)[a] F 1, R 1)[b]	5’- TTCTTCTACATCATCTCCCTCTAACCTCCTAT5fCGTA5fCGTTA5fC GATA5fC GTTAGGTAGATTAGAGAGTAGTAGTGGTGAGGA-3’ Complementary sequence 3’- AAGAAGATGTAGTAGAGGGAGATTGGAGGATAG5fCATG5fCAATG5fCTATG5fCAATCCATCTAATCTCTCATCATCACCACTCCT -5’
80 bp ds ODN-C-2	5’- CTAAATCTACTAAATCCTCTAAATCTATTCTATACATTAATATTAATTAAAGGTAGTAGTAGTAGATATAAGATGATAGG-3’ Complementary sequence 3’- GATTTAGATGATTTAGGAGATTTAGATAAGATATGTAATTATAATTAATTTCCATCATCATCATCTATATTCTACTATCC-5’
E-DNA-fC (Template 6)[a] F 2, R 2)[b]	5’- TCCTCCTACATCATTCCTCTCTAACCCCTTATATGTA5fCTTAGAA T5fCAATTGAGTGATTGAAGGTAGTTAGTGGTGGTAGA -3’ Complementary sequence 3’- AGGAGGATGTAGTAAGGAGAGATTGGGGAATATA5fCATGAAT5fCTTAGTTAACTCACTAACTTCCATCAATCACCACCATCT -5’
E-DNA-C (Template 6)[a] F 2, R 2)[b]	5’- TCCTCCTACATCATTCCTCTCTAACCCCTTATATGTACTTAGAATCAATTGAGTGATTGAAGGTAGTTAGTGGTGGTAGA -3’ Complementary sequence 3’- AGGAGGATGTAGTAAGGAGAGATTGGGGAATATACATGAATCTTAGTTAACTCACTAACTTCCATCAATCACCACCATCT -5’
E-DNA-mC (Template 6)[a] F 2, R 2)[b]	5’- TCCTCCTACATCATTCCTCTCTAACCCCTTATATGTA5mCTTAGAA T5mCAATTGAGTGATTGAAGGTAGTTAGTGGTGGTAGA -3’ Complementary sequence 3’- AGGAGGATGTAGTAAGGAGAGATTGGGGAATATA5mCATGAAT5mCTTAGTTAACTCACTAACTTCCATCAATCACCACCATCT -5’
E-DNA-hmC (Template 6)[a] F 2, R 2)[b]	5’- TCCTCCTACATCATTCCTCTCTAACCCCTTATATGTA5hmCTTAGAAT5hmCAATTGAGTGATTGAAGGTAGTTAGTGGTGGTAGA -3’ Complementary sequence 3’- AGGAGGATGTAGTAAGGAGAGATTGGGGAATATA5hmCATGAAT5hmCTTAGTTAACTCACTAACTTCCATCAATCACCACCATCT -5’
seq-1 ds-ODN-fC	5’- CTAAATCTACTAAATCCTCTAAATCTATTCTATA5fCATTAATATTAATTAAAGGTAGTAGTAGTAGATATAAGATGATAGG-3’

	Complementary sequence 3'-<br>GATTTAGATGATTTAGGAGATTTAGATAAGATATGTAATTATA<br>ATTAATTTCCATCATCATCATCTATATTCTACTATCC-5'
seq-2 ds-<br>ODN-fC	5'-<br>CTAAATCTACTAAATCCTCTAAATCTATTCTATAAATTAAT5fCT<br>TAATTAAAGGTAGTAGTAGTAGATATAAGATGATAGG-3'<br>Complementary sequence 3'-<br>GATTTAGATGATTTAGGAGATTTAGATAAGATATTTAATTAGA<br>ATTAATTTCCATCATCATCATCTATATTCTACTATCC-5'
seq-3 ds-<br>ODN-fC	5'-<br>TTCTTCTACATCATCTCCCTCTAACCTCCTATATGTA5fCTTAG<br>AAT5fCAATTAGGTAGATTAGAGAGTAGTAGTGGTGAGGA-3'<br>Complementary sequence 3'-<br>AAGAAGATGTAGTAGAGGGAGATTGGAGGATATAC ATGAAT<br>CTTAGTTAATCCATCTAATCTCTCATCATCACCACTCCT -5'

11. The templates used for synthesis of dsDNA

Table S2. The sequences of the templates used in this study.

ODNs	Sequence (5'-3')
Template 1	TTCTTCTACATCATCTCCCTCTAACCTCCTATATGTACTTAGAATC<br>A<br>ATTAGGTAGATTAGAGAGTAGTAGTGGTGAGGA
Template 2	TTCTTCTACATCATCTCCCTCTAACCTCCTATATTACGTTATAATA<br>AA<br>TTAGGTAGATTAGAGAGTAGTAGTGGTGAGGA
Template 3	TTCTTCTACATCATCTCCCTCTAACCTCCTATATTACGTTACGATA<br>AA<br>TTAGGTAGATTAGAGAGTAGTAGTGGTGAGGA
Template 4	TTCTTCTACATCATCTCCCTCTAACCTCCTATATTACGTTACGATA<br>CG<br>TTAGGTAGATTAGAGAGTAGTAGTGGTGAGGA
Template 5	TTCTTCTACATCATCTCCCTCTAACCTCCTATCGTACGTTACGATA<br>CG<br>TTAGGTAGATTAGAGAGTAGTAGTGGTGAGGA
Template 6	TCCTCCTACATCATTCCTCTCTAACCCCTTATATGTACTTAGAATC<br>A<br>ATTGAGTGATTGAAGGTAGTTAGTGGTGGTAGA

12. The sequences of primers used in this study

Table S3. The sequences of the primers used in this study.

ODNs	Sequence (5'-3')
Forward primer 1	TTCTTCTACATCATCTCCCTCTAACCTCCT
Reverse primer 1	TCCTCACCACTACTACTCTCTAATCTACCT

Forward primer 2	TCCTCCTACATCATTCCTCTCTAACCCCT
Reverse primer 2	TCTACCACCACTAACTACCTTCAATCACTC
Chr5-F primer	GTTGACGGCTGGATTAGAATG
Chr5-R primer	AGAACCGGTGTTCTAAAGCT
Chr14-F primer	GCCTGCTATACAATTCACGC
Chr14-R primer	CCTTGTTTTACCCAGGGGAT
Chr17-F primer	CTTGTCGGTTCTTCGGACT
Chr17-R primer	CATTGGGCGTTACTGTCATC
Chr18-F primer	GTCAAGAACGTGGTTGAAGG
Chr18-R primer	ACTTTAGCTGCTTGACCAGT

Reference

[1] O. Demeter, E. A. Fodor, M. Kállay, G. Mező, K. Németh, P. T. Szabó, P. Kele, *Chem. E. J.* **2016**, *22*, 6382.

[2] C. Yang, J. Mi, Y. Feng, L. Ngo, T. Gao, L. Yan, Y. G. Zheng, *J. Am. Chem. Soc.* **2013**, *135*, 7791-7794.
